# The Short Synacthen Test and Its Utility in Assessing Recovery of Adrenal Function in Patients With Central Adrenal Insufficiency

**DOI:** 10.1210/jc.2018-01317

**Published:** 2018-08-03

**Authors:** Mark Sherlock, Paul M Stewart

**Affiliations:** 1 Department of Endocrinology, Beaumont Hospital, Beaumont, Dublin 9, Ireland; 2 Department of Endocrinology, Royal College of Surgeons in Ireland Medical School, Beaumont, Dublin 9, Ireland; 3 Medical School, University of Leeds, Leeds, United Kingdom

## Abstract

The short synacthen test is useful in assessing recovery of adrenal function in patients with central adrenal insufficiency.

Adrenal insufficiency (AI) is classified according to a primary adrenal cause or as a consequence of central AI [which can be divided into secondary AI (SAI) and tertiary AI (TAI)] (1). SAI is caused by the loss of corticotroph cell function, most frequently by pituitary tumors and/or their subsequent treatment including surgery and radiotherapy (2). By contrast, TAI occurs due to suppression of corticotroph function following exogenous glucocorticoid (GC) therapy and is a major unmet clinical need with 0.5% to 2% of the population taking exogenous GC therapy to treat a variety of inflammatory diseases (3). Hypothalamic-pituitary-adrenal axis suppression by exogenous GCs has been associated with adrenal crisis in patients treated with systemic, inhaled, intra-articular and topical GCs (4, 5) and is related to the cumulative exposure (determined by a combination of the duration of therapy and the dose and relative potency of GC used). However, there is considerable interindividual variability in the response to GCs and as such any consensus to what defines the level of exposure required to cause AI is difficult.

The short synacthen test (SST) is now the most widely used method of assessment of adrenal sufficiency. Tetracosactrin, a synthesized polypeptide with an amino acid sequence including 1 to 24 of the 1 to 39 chain of the naturally occurring corticotropin, has adrenocortical stimulatory action equivalent to that of natural corticotropin (6). With adrenal reserve post exogenous ACTH dictated by the prevailing level of endogenous ACTH, a number of studies have shown that the SST performs well when compared with the originally described “gold standard” insulin tolerance test (ITT) in assessing AI/sufficiency (7, 8). However, it is important to note that such studies have predominantly assessed GC sufficiency in an at-risk population of AI rather than recovery of adrenal function in patients with established AI. In the former studies peak cortisol pass/fail values across an SST have been validated against an ITT; basal random cortisol concentrations and incremental change post-SST have been unhelpful discriminators.

Here the study by Pofi *et al.* (9) is a useful addition to the literature reporting results of synacthen testing from a large retrospectively studied cohort of patients with central AI, comprising both SAI (n = 776) and TAI (n = 110), from three academic endocrine centers. It is important to make the distinction between patients with SAI and TAI in this study as the potential for recovery of adrenal function (and thus pre-test probability of recovery) are significantly different in each group but overall across the entire cohort of 886 patients, 37% of patients who initially failed an SST eventually went on to subsequently pass an SST.

In the pituitary disease group of patients with SAI, 57% of patients with nonfunctioning pituitary tumors and 44% of patients who underwent pituitary surgery subsequently passed the SST. This is significantly higher than one would have expected and has major potential implications for clinical practice. Although untreated or unrecognized AI confers the risk of adrenal crisis and increased morbidity and mortality, it has also become increasingly evident that morbidity and mortality are increased in patients with AI taking replacement GC (10–16). The mortality excess is largely due to increased cardiovascular deaths (15), most likely from subtle but prolonged increase in either dose of GC replacement and/or a noncircadian mode of replacement, effectively leading to mild Cushing syndrome (17). Impaired quality of life is a further major issue that again appears to be related to AI but also GC exposure (18, 19). The realization that many patients with established SAI might recover endogenous adrenal function and thereby avoid lifelong GC replacement is clearly important.

In patients with SAI, the 30-minute cortisol response post-SST was the best indicator of future (and more prompt) adrenal recovery, but accuracy of predicting future recovery of adrenal function was further enhanced by combining the 30-minute cortisol SST value with the baseline cortisol (which can be taken as being equivalent to a random morning cortisol). In the TAI group it was the delta cortisol across the SST (the difference between the baseline and 30-minute sample) that was the best predictor for future adrenal recovery. Combining the delta cortisol threshold with a subsequent random cortisol (above or below 200 nmol/L) helped to refine the predictive ability of the test.

The paper has some limitations, which the authors highlight, including the retrospective nature of the study, potential selection bias, and lack of data regarding GC exposure.

The diagnostic cutoffs of a “normal” cortisol response to dynamic testing including SST have been debated for some time. The ITT “pass” value was originally defined as the minimum cortisol response required to safely undergo a stressful event (elective surgery) (20). With the emergence of immunoassays, it was found that the previously used fluorometric assays overestimated the cortisol response to synacthen due to lack of assay specificity and cross-reactivity with other GCs (corticosterone and cortisone). The advent of modern radioimmunoassay and the demonstration of a close correlation between the ITT and the SST further reinforced the view that the commonest thresholds used to indicate normal function should be approximately >500 nmol/L or 550 nmol/L (8, 21) and with newer assays as low as 450 nmol/L (22). This study uses three different assays (including two different generations of the same assay system), which have significantly different cutoffs for passing the SST and although the authors have attempted to control for this, it remains a potential factor that should be taken into account when interpreting the data. Importantly, when assessing any cutoff for the SST, the assay used and assay specific cutoff for cortisol on an SST must be defined. There is substantial interassay variation in cortisol assays; Klose *et al.* (22) and more recently El-Farhan *et al.* (23) demonstrated that individual samples may differ by up to 50 to 110 nmol/L when measured by different assays. Similarly, Clarke *et al.* (24) have shown substantial gender differences in healthy controls when assessing cortisol response to SST depending on the assay used.

As well as the utility of the peak cortisol value post-SST, the authors highlight the importance of the delta cortisol to predict future recovery of AI. It is perhaps not surprising that in a group of patients with suppressed adrenal function post exogenous GC therapy as opposed to a “normal” population being evaluated for adrenal sufficiency, the incremental change in cortisol was clinically useful. However, it is important to stress that the SST in this context has yet to be validated against the ITT; Kane *et al.* (25) in a small series of GC treated rheumatology patients highlighted differences between the performance of the SST and ITT in patients with TAI; 8/22 patients failing the SST but passed the ITT.

Another key factor in interpreting this data are that patients with hypopituitarism and pituitary tumors are a very heterogenous group with differences in tumor size, tumor invasiveness, degree of surgical intervention, degree of hypopituitarism, and dose of hydrocortisone therapy prescribed. All of these factors may impact on the possibility of subsequent recovery of adrenal function; for example, if ACTH was the only deficiency and there was no gonadotropin or GH deficiency, one might be suspicious that this was not a true initial SST fail, given the hierarchy of pituitary hormone deficits that is often encountered. Clinical data are lacking on the characteristics of the SAI group; more studies are required with greater clinical details before generalizing to other populations.

The significant rates of recovery of adrenal function in the pituitary patient group is intriguing. Arafah *et al.* (26) initially studied this concept in 1994 and postulated that recovery in AI following pituitary surgery could be due to a decrease in compression of the portal vessels leading to an improved delivery of hypothalamic hormones. Although there have been reports of recovery of hypothalamic-pituitary-adrenal function in SAI, the percent recovery rate here is surprising. Munro *et al.* (27), albeit in a smaller cohort, reported recovery of SAI in 17.6% during follow-up. There may be a potential selection bias; it would not be routine practice to repeat dynamic testing in patients labeled with SAI without additional indication that recovery was possible. It is not clear what the indications for this repeat testing were in this particular study. Similarly, the authors advocate that patients with SAI who have a 30-minute cortisol >350 nmol/L should have repeat testing every 6 months. In contrast, the <350 nmol/L cohort had a much slower recovery and an annual testing strategy could be adopted. There may be bias here, in that the timing of the repeat testing was left to individual clinicians’ discretion and was not part of a protocolized pathway, and as such patients who had lower 30-minute values may not have been tested as frequently or early.

Despite these limitations the use of the peak cortisol value post-SST with or without the additional predictive value of a basal cortisol sample and incremental response during SST may be very helpful in predicting the chance of future recovery of adrenal function. This should encourage a lower threshold for further retesting in patients with both SAI and TAI but may also prevent unnecessary SSTs when there is little chance that the patient will pass a subsequent SST. This study will need to be supported by prospective data but raises important clinical practice issues, particularly given the GC-related increased morbidity and mortality of patients with SAI and TAI.

## Abbreviations:

AI: adrenal insufficiency
GC: glucocorticoid
ITT: insulin tolerance test
SAI: secondary adrenal insufficiency
SST: short synacthen test
TAI: tertiary adrenal insufficiency
